# Exploring psychological impact of hand-arm vibration syndrome (HAVS)

**DOI:** 10.1093/occmed/kqae138

**Published:** 2025-02-10

**Authors:** S Bezanson, A Ruco, M Howse, D Budd, R House, M Khakpour, D L Holness

**Affiliations:** Interdisicplinary Health Program, St. Francis Xavier University, Antigonish, Nova Scotia, Canada; Interdisicplinary Health Program, St. Francis Xavier University, Antigonish, Nova Scotia, Canada; Peter Gilgan Centre for Women’s Cancers, Women’s College Hospital, Toronto, Ontario, Canada; VHA Home HealthCare, Toronto, Ontario, Canada; Research, Nova Scotia Health, Halifax, Nova Scotia, Canada; Interdisicplinary Health Program, St. Francis Xavier University, Antigonish, Nova Scotia, Canada; Centre for Urban Health Solutions, Li Ka Shing Knowledge Institute, St. Michael’s Hospital, Unity Health Toronto, Toronto, Ontario, Canada; Division of Occupational Medicine, Department of Medicine, St. Michael’s Hospital, Unity Health Toronto, Toronto, Ontario, Canada; Dalla Lana School of Public Health and Department of Medicine, University of Toronto, Toronto, Ontario, Canada; Division of Occupational Medicine, Department of Medicine, University of Toronto, Toronto, Ontario, Canada; Interdisicplinary Health Program, St. Francis Xavier University, Antigonish, Nova Scotia, Canada; Centre for Urban Health Solutions, Li Ka Shing Knowledge Institute, St. Michael’s Hospital, Unity Health Toronto, Toronto, Ontario, Canada; Division of Occupational Medicine, Department of Medicine, St. Michael’s Hospital, Unity Health Toronto, Toronto, Ontario, Canada; Dalla Lana School of Public Health and Department of Medicine, University of Toronto, Toronto, Ontario, Canada; Division of Occupational Medicine, Department of Medicine, University of Toronto, Toronto, Ontario, Canada

## Abstract

**Background:**

Hand-arm vibration syndrome (HAVS) is an occupational disease associated with long-term exposure to power tools leading to hand-transmitted vibration exposure. Prior research has focussed on physical manifestations with little known about the psychological impacts of HAVS.

**Aims:**

To examine if HAVS severity and/or functional impairment is associated with psychological outcomes.

**Methods:**

We conducted a cross-sectional study collecting data through a survey and retrospective chart review of workers being assessed for HAVS at an occupational medicine clinic. We collected information on demographics, work conditions, disease characteristics and physical and psychological outcomes as measured through validated instruments (SF-12, QuickDASH, GAD-2, PHQ-2). Descriptive statistics and bivariate analyses were followed by multivariable models to explore associations between mental health outcomes and predictor variables.

**Results:**

Participants (*N* = 94; 56% response rate) were male with a mean age of 48.2 years. The majority (62%) worked in the mining sector, and 27% of participants reported feeling depressed and 35% reported showing little interest in or pleasure in doing things, while 28% reported clinically significant anxiety symptoms. In multivariable models, the QuickDASH, a measure of upper-extremity function and disability, was the only significant predictor of psychological outcomes.

**Conclusions:**

Workers with HAVS have poorer mental health and physical functioning outcomes in comparison to the general population. Employers should consider tailored policies and interventions to address the mental health of workers with HAVS.

## Teaser

This paper explores the psychological outcomes of workers with hand-arm vibration syndrome (HAVS) who were assessed at an occupational medicine clinic. Most participants worked in the mining and construction sector. Various psychological outcomes were common in workers with HAVS. In multivariable models, the QuickDASH, a measure of upper-extremity function and disability, was the only significant predictor of psychological outcomes. Tailored interventions to address the mental health of workers with HAVS may be needed.

## Introduction

Hand-arm vibration syndrome (HAVS) is an occupational disease associated with long-term use of hand-held vibrating tools and equipment. It is most common in manual occupations such as mining, construction, forestry, and manufacturing [1, 2]. The physical manifestations and functional impairments associated with HAVS have been relatively well studied [1, 3–5]. However, less is known about the psychological impacts of HAVS. House et al. [6] reported that the SF-12 mental quality of life scores in Canadian workers with HAVS were significantly lower than the normal values for the general population. Qualitative studies cite loss of identity, poor self-perception, embarrassment, withdrawal from social activities, loss of independence, anger and anxiety as common psychological effects of HAVS [4, 7, 8]. In our study, we examined the hypothesis that HAVS severity and/or functional impairment is associated with poorer psychological outcomes.

**Key learning points**

**What is already known about this subject:**

- Hand-arm vibration syndrome (HAVS) is an occupational disease associated with long-term use of hand-held vibrating tools and equipment and is common among manual labour workers.
- Physical manifestations of the disease include changes related to vascular, neurological and musculoskeletal functions in the fingers, hands and upper extremities such as numbness and tingling, blanching, increased cold sensitivity, decreased motor control and reduced grip strength.

**What this study adds:**

- Workers with HAVS have poorer mental health in comparison to the general population with over a quarter reporting feeling depressed.
- QuickDASH score, a measure of upper-extremity function and disability, was a significant predictor of mental health outcomes.

**What impact this may have on practice or policy:**

- The QuickDASH may be a useful, rapid screening tool to identify workers with HAVS-related disability and increased likelihood of mental health outcomes.
- Tailored workplace policies, interventions and supports are needed to prevent HAVS and to address not only the physical but also the psychological impact of HAVS.

## Methods

This cross-sectional study collected data through a survey and retrospective chart review of workers assessed for HAVS at a specialist occupational medicine clinic in Toronto, Canada. All of the patients had been referred by the workers’ compensation board of Ontario for assessment of HAVS. The St. Michael’s Hospital Research Ethics Board approved the study. A total of 169 patients were asked to participate, but only 95 (56%) consented and completed the survey. One patient was female and was excluded from the analyses, leaving 94 patients (56%) in the final study group.

Participants filled out a survey that included the following validated instruments: SF-12 (12-Item Short Form Health Survey) [9], GAD-2 (Generalized Anxiety Disorder Scale) [10], QuickDASH [11] and a modified version of the PHQ-2 (Patient Health Questionnaire-2) [12], which is used to screen for depression, in addition to information about work conditions and demographics. Data extracted from patient charts included variables related to disease characteristics including HAVS sensorineural and vascular staging (Stockholm scale) and grip strength. The person carrying out the chart abstraction (DB) was blinded to the results of the survey. Descriptive statistics were used to summarize the study sample including work history and disease characteristics stratified by sector (mining versus construction) and age category (<50 years versus 50+ years), and Chi-square tests or *t*-tests to explore bivariate associations. Multivariable analyses for the outcomes of the GAD-2 and the SF-12 Mental Component Score were also explored through binary logistic regression and multiple linear regression, respectively. We used a GAD-2 score of ≥3 to indicate clinically significant anxiety. We explored both saturated models adjusted for key predictor variables and covariates (QuickDASH, SF-12 Physical Component Score, right grip strength, sensorineural and vascular Stockholm staging scores, age category, sector, working status (currently working or not) and years experiencing symptoms) and simpler models where only variables that demonstrated a significant or marginally significant association with the outcome of interest were included.

## Results

Participants had a mean age of 48.2 years (range 26–69 years), and the majority worked in mining (62%) and construction (32%). 27% reported feeling depressed and 35% reported showing little interest in doing things. Clinically significant anxiety symptoms were detected among 28% of participants. Table 1 presents disease and work characteristics, along with psychological and physical functioning outcomes within sector and age subsets, and highlights significant group differences. In the bivariate analysis, the QuickDASH score and SF-12 physical component score were associated with both the GAD-2 and SF-12 psychological outcomes, and the Stockholm vascular stage was also associated with the GAD-2. Multivariable analysis indicated that in all models, including both simple and saturated models, the QuickDASH score was the only significant predictor for mental health outcomes. Table 2 presents the results when only significant factors from the bivariate analysis were included in the models.

**Table 1.** Prevalence of disease and work characteristics, and psychological and physical functioning outcomes by sector and age

		Construction (*N* = 30) *N* (%)	Mining (*N* = 59) *N* (%)	Total (*N* = 89) *N* (%)	<50 years old (*N* = 47) *N* (%)	50 + years old (*N* = 47) *N* (%)	Total (*N* = 94) *N* (%)
**Disease characteristics**							
Years experiencing HAVS symptoms	<5	6 (20)	27 (46)	33 (37)	24 (51)	11 (23)	35 (37) ^**^
	5–10	9 (30)	13 (22)	22 (25)	14 (30)	10 (21)	24 (26)
	>10	12 (40)	18 (31)	30 (34)	8 (17)	23 (49)	31 (33)
Sensorineural Staging (Stockholm Scale)	Severe symptoms (scores > 2)	6 (20)	15 (26)	21 (24)	10 (21)	14 (30)	24 (26)
Vascular Staging (Stockholm Scale)	Severe symptoms (scores ≥ 2)	12 (40)	36 (61)	48 (54)	26 (55)	25 (53)	51 (54)
Right hand grip^‡^ strength (kg)	≤37	13 (43)	18 (31)	31 (35)	5 (11)	25 (53)	30 (32)^***^
	>37 to 45.3	5 (17)	23 (40)	28 (32)	20 (44)	14 (30)	34 (37)
	>45.3	12 (40)	17 (29)	29 (33)	21 (46)	8 (17)	29 (31)
**Work characteristics**							
Currently working	No	8 (27)	16 (27)	24 (27)	10 (21)	15 (32)	25 (27)
	Yes	21 (70)	42 (71)	63 (71)	37 (79)	30 (64)	67 (71)
If not, because of symptoms		5 (63)	10 (63)	15 (63)	7 (70)	9 (60)	16 (17)
Work outdoors	No	2 (7)	34 (58)	36 (40)^***^	23 (49)	15 (32)	38 (40)
	Yes	25 (83)	17 (29)	42 (47)	19 (40)	26 (55)	45 (48)
Exposed to hand vibration	No	1 (3)	6 (10)	7 (8)	2 (4)	5 (11)	7 (7)
	Yes	24 (80)	49 (83)	73 (82)	43 (92)	35 (75)	78 (83)
Exposed to foot vibration	No	8 (27)	13 (22)	21 (24)	8 (17)	15 (32)	23 (25)
	Yes	15 (50)	41 (70)	56 (63)	34 (72)	24 (51)	58 (62)
**Outcomes**							
Stress	No	17 (57)	35 (59)	52 (58)	27 (57)	25 (53)	52 (55)
	Yes	11 (37)	14 (24)	25 (28)	16 (34)	13 (28)	29 (31)
Depressed^+^ (PHQ-2)	No	22 (73)	41 (70)	63 (71)	33 (70)	31 (66)	64 (68)
	Yes	6 (20)	15 (25)	21 (24)	14 (30)	11 (23)	25 (27)
Little interest^+^ (PHQ-2)	No	22 (73)	33 (56)	55 (62)	30 (64)	26 (55)	56 (60)
	Yes	6 (20)	23 (39)	29 (33)	17 (36)	16 (34)	33 (35)
GAD-2 score	<3	20 (71)	45 (79)	65 (77)	37 (41)	28 (31)	65 (72)
	≤3	8 (29)	12 (21)	20 (24)	10 (11)	15 (17)	25 (28)
SF-12 Mental Component Score		**Mean (SD)**	**Mean (SD)**	**Mean (SD)**	**Mean (SD)**	**Mean (SD)**	**Mean (SD)**
		50.6 (9.5)	49.4 (9.5)	49.9 (9.5)	49.6 (9.2)	49.2 (10.3)	49.4 (9.6)
SF-12 Physical Component Score		36.6 (8.3)	41.2 (7.4)	39.6 (8.0)	42.0 (7.6)	36.6 (7.5)	39.5 (8.0)
QuickDASH Score		44.5 (17.0)	42.8 (18.2)	43.3 (17.8)	38.5 (17.4)	50.2 (16.3)	44.1 (17.8)^**^

^**^ *P*-value < 0.01

^***^ *P*-value < 0.001.

^‡^Grip strength categories are based on a tertile split.

^+^For modified PHQ-2 items, participants reported ‘No’ if symptom was absent and ‘Yes’ if symptom was present.

**Table 2.** Multivariable models for outcomes of GAD-2 and SF12—Mental Component Score^*^

(a)Logistic regress with GAD-2 as the dependent variable										
Parameter	B		Standard error		Exp(B)		*P*-value			
Intercept	−7.596		4.3006		<0.001		NS^**^			
QuickDASH score	0.127		0.0416		1.136		<0.01			
SF-12 Physical Component score	−0.010		0.0650		0.990		NS			
Vascular score	0.719		0.7946		2.052		NS			
**(b) Multiple linear regression with SF12—Mental Component Score as the dependent variable**										

Parameter		Standardized coefficients beta		Standard error		*P*-value		*R*	*R* ^2^	
QuickDASH score		−0.534		0.068		<0.001		0.455	0.207	
SF-12 Physical Component Score		−0.188		0.155		NS				

^*^Only those variables from the bivariate analysis that were associated with the dependent variable of interest were included in the models.

^**^NS = not statistically significant.

## Discussion

Our results suggest that many workers with HAVS experience poor mental health outcomes. Participants in our study commonly reported feeling depressed and having little interest in doing things, as well as having clinically significant anxiety symptoms. The key predictor of poorer psychological outcomes, including the SF-12 mental component and GAD-2 scores, was the QuickDASH score.

Strengths of our study included collecting excellent data from both a detailed survey and clinical chart abstraction, as well as the use of validated instruments to assess clinically important outcomes. Though we had an acceptable response rate, our study had a relatively small sample size with most of the sample limited to workers in the construction and mining sectors. This was a cross-sectional study with no pre-morbid psychological assessments, and therefore, the direction of causation can be questioned. This is especially the case in studies of psychological factors and disability. For example, previous work has shown that psychological factors in combination with musculoskeletal pain may enhance reported disability [13].

The SF-12—Mental Component Score in our sample was similar to that reported in a previous study of workers with HAVS [6], and both were lower (indicating worse mental health) than the average scores for the Canadian general population [14]. The impact of HAVS on decreased mental health is similar to such effects due to other occupational conditions. For example, occupational musculoskeletal conditions [15], psychosocial stress at work [16], anxiety related to occupational exposure to asbestos [17] and being unemployed [18] have all been shown to be associated with similar low SF-12 scores.

The average total score (±SD) for the QuickDASH was 44.1 (±17.8) in our study, slightly higher than mean scores for the full DASH instrument among workers with HAVS as reported by House et al. (42.2 ± 20.9) [19]. The QuickDASH scores reported in our sample were also much higher, indicating greater disability, than average scores for the US general population [20]. Previous investigation has found that HAVS vascular, sensorineural, and musculoskeletal effects are independently associated with increased upper extremity disability as measured by the DASH questionnaire [19].

More attention needs to be paid to the psychological effects of HAVS by workers, their health care providers and employers. The results of our study suggest that the QuickDASH may be a useful, rapid screening tool to identify workers with HAVS-related disability and increased likelihood of psychological outcomes.
